# Enhanced electron-phonon coupling for a semiconductor charge qubit in a surface phonon cavity

**DOI:** 10.1038/srep15176

**Published:** 2015-10-15

**Authors:** J. C. H. Chen, Y. Sato, R. Kosaka, M. Hashisaka, K. Muraki, T. Fujisawa

**Affiliations:** 1 Department of Physics, Tokyo Institute of Technology, 2-12-1 Ookayama, Meguro, 152-8551, Japan; 2 NTT Basic Research Laboratories, NTT Corporation, 3-1 Morinosato-Wakamiya, Atsugi, 243-0198, Japan

## Abstract

Electron-phonon coupling is a major decoherence mechanism, which often causes scattering and energy dissipation in semiconductor electronic systems. However, this electron-phonon coupling may be used in a positive way for reaching the strong or ultra-strong coupling regime in an acoustic version of the cavity quantum electrodynamic system. Here we propose and demonstrate a phonon cavity for surface acoustic waves, which is made of periodic metal fingers that constitute Bragg reflectors on a GaAs/AlGaAs heterostructure. Phonon band gap and cavity phonon modes are identified by frequency, time and spatially resolved measurements of the piezoelectric potential. Tunneling spectroscopy on a double quantum dot indicates the enhancement of phonon assisted transitions in a charge qubit. This encourages studying of acoustic cavity quantum electrodynamics with surface phonons.

Coupling of electrons to intrinsic phonons has been known to cause scattering^1^ or energy relaxation^2^,^3^,^4^ in semiconductor electronic systems. Previous studies^2^,^3^ showed that spontaneous emission of energies into the phonon bath is a major dissipation mechanism in charge and spin qubits in GaAs/AlGaAs systems. However, this negative effect can be applied in a constructive way to realise an acoustic analog of cavity quantum electrodynamic (cQED) system^5^,^6^,^7^,^8^ for reaching the strong or ultra-strong coupling regime in cQED. It will be advantageous to fabricate an acoustic cQED system because it traps phonons with wavelengths in the nanometer regime, which is smaller than the microwave cavities used in conventional cQED systems. Smaller wavelengths allow for the fabrication of a smaller cavity with the potential of achieving a larger electron-cavity coupling^9^. Previously phonon cavities have been demonstrated on suspended nanowires^10^,^11^,^12^,^13^ however it is challenging to confine intrinsic crystallographic phonons^6^,^7^,^8^. Acoustic waves in piezoelectric materials, such as GaAs, has a large coupling to quantum dots^14^,^15^,^16^,^17^ or superconducting qubits^18^,^19^, and are advantageous for realising strong or ultra-strong coupling regimes with a small cavity^9^.

In this study we demonstrate phonon cavities for surface acoustic waves (SAW) on a GaAs/AlGaAs heterostructure. Brag reflectors (BR) deposited onto the surface of the heterostructure are used as mirrors to reflect and trap SAW. By observing the piezoelectric potential of the SAW through time, spatial and frequency measurements, we identify the phonon band gap and cavity modes of our phonon cavities. Furthermore coupling of the cavity mode to a charge qubit is demonstrated through phonon assisted transport measurements on a double quantum dot and observe to be enhanced at the resonant frequency of the cavity.

The coupling frequency *g* between a qubit and a cavity mode can be enhanced by reducing the mode volume *V* of the cavity. For standard cQED with electromagnetic waves, *g* normalized by the resonant frequency *ω* is bounded by the fine structure constant α ~ 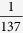, as seen in the form 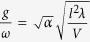 for an electric dipole of the length *l* and the wavelength *λ*^5^,^20^. Since geometric constraints require *V* to be greater than *l*^2^*λ*, 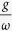 is practically limited by 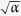. The phonon cavity is attractive for overcoming this limitation by taking advantage of its small mode volume associated with the slow speed *v* compared with the light speed *c*. Considering the electromechanical coupling *K*^2^ (0.07% for GaAs SAW)^21^,^22^, the normalized coupling for acoustic cQED is bounded by the effective fine structure constant 
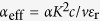
 (*ε*_r_ being the relative dielectric constant). This can be greater than *α* in some piezoelectric materials (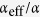 ~ 10 for GaAs SAW and potentially greater than 100 for ZnO SAW and other piezoelectric materials^23^), and thus attractive for reaching the ultra-strong coupling regime. We show that GaAs SAW phonons are successfully confined in a cavity for enhancing the electron-phonon coupling.

The coupling between SAW and a charge qubit in two-dimensional electron gas (2DEG) of a GaAs/AlGaAs heterostructure is significant even without forming a cavity^24^. SAW is a Rayleigh wave localized near the surface within the penetration length (≈0.3 *λ*) comparable to the SAW wavelength *λ*, which provides a large piezoelectric field at the 2DEG located near the surface^25^. A charge qubit, in which an excess electron occupies one of the two dots, has an electric dipole (*el*) for the dot distance *l*^26^. The coupling can be maximized when *l* is comparable to *λ*/2 of SAW that resonate with the level spacing of the qubit (typically a few GHz)^2^. Typical GaAs DQDs approximately meet this condition (*λ* = 800 nm for the SAW frequency *f* = 3.2 GHz, 2DEG depth *d* = 95 nm and *l* = 240 nm in our device). We show that SAW can be confined in a cavity and the coupling can be further enhanced by designing a SAW cavity.

A SAW cavity can be formed by two BRs made of periodic metal arrays with a period *a*, shown by the schematic in Fig. 1a. Metallization of the semiconductor surface decreases the SAW velocity by about 10% and causes a small reflection with a coefficient *r* (≈1%) at the metal edges^27^. With a large number *N*_BR_ (≳ 1/*r*) of the array, the total reflection coefficient approaches unity for a specific wavelength *λ*_BR_ close to 2*a*. When two BRs are separated by a gap distance *D* which equals an odd integer multiples of *λ*_BR_/2, the fundamental cavity mode exhibits a node at the center, which maximizes the coupling to the DQD, as shown schematically in Fig. 1a.

The important parameter of the cavity for cQED is the finesse *F*, which determines the number of times the wave is reflected inside the cavity before decaying^20^. Therefore, the coupling is enhanced by the factor *F*. We experimentally evaluate the enhancement by the following schemes. The time-resolved reflectometry reveals each reflected wavepacket to characterize the phonon band gap inside the BR. The finesse is evaluated as a ratio of the band gap to the resonance width of the cavity mode. The obtained *F* ~ 80 is promising for cQED applications. Moreover, the SAW in the cavity mode induces phonon-assisted qubit transitions more efficiently than off-resonant SAW, which manifests the enhancement of electron-phonon coupling in the system.

## Results and Discussions

In our experiments, SAW phonons are excited by applying an RF voltage to an interdigital transducer (IDT) sharing the metal array with the BR, and the enhancement of the piezoelectric potential is evaluated with electronic devices inside the cavity. Device A illustrated in Fig. 2a consists of two metal gratings with *a* = 0.4 *μ*m (*λ*_BR_ = 0.8 *μ*m) which serve as two BRs and an IDT shared with the left BR. A short SAW packet launched from the IDT bounces between the two BRs with a large distance 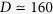 *μ*m. The time resolved phase-sensitive local potential measurements were performed with a point contact (PC), where *i*-th PC (*i* = 1−5) is selectively activated by using gates G_0_ and G_*i*_^28^,^29^. The measurement was done by applying two synchronized burst waves, *V*_ex_ for generating a wave packet and *V*_det_ for detecting the potential wave 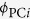 at *i*-th PC, and by measuring the resultant dc current *I*_det_ through the PC as a function of the delay time *t*_d_ between the two bursts [See pulse sequence in Fig. 2b and Methods]. The PCs separated by 0.12 *μ*m (0.15*λ*) allow us to identify the phase evolution and thus the direction of the travelling waves.

Figure 2c shows the derivative of the detection current 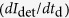 with PC2 as a function of *t*_d_, which oscillates with a period (1/*f* ~ 0.3 ns) of the carrier frequency *f* = 3.2 GHz as seen in the insets. The time evolution of the envelope in the main trace involves the electromagnetic (EM) crosstalk with a triangular shape centered at *t*_d_ = 0, direct SAW packet at around *t*_d_ = 31 ns (d-SAW), and other reflected waves (r1- and r2-SAW). Their travelling-wave nature is confirmed from the phase evolution of the signals at different PCs. For the direct SAW packet in the left inset of Fig. 2c, the phase of the oscillations measured at the rightmost PC4 is seen to be delayed with respect to that at the leftmost PC1, indicating that the wave packet is propagating to the right. Similarly, examining the phase evolutions around *t*_d_ ≈ 99 ns (the central inset) and *t*_d_ ≈ 142 ns (the right inset) allows us to assign the oscillations in these time domains to be the primary reflected waves moving to the left (r1-SAW) and the secondary reflected waves moving to the right (r2-SAW), respectively. Observation of SAW packets bouncing between BRs suggests high coherency of SAW even in the presence of electronic devices with fine gate patterns G_0−5_.

The reflection spectrum of a BR is obtained by plotting the amplitude *A*_det_ of the oscillation in 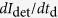 around some specific delay time *t*_d_ as a function of *f* in Fig. 2e. The broad spectrum of the direct SAW at *t*_d_ = 35 ns, which is determined by the Fourier spectrum of the SAW burst wave. This is regarded as an incident spectrum to the right BR. The reflected spectrum measured at a later time *t*_d_ > 60 ns is narrower and the width is independent of *t*_d_. This is a manifestation of the phonon band gap in which SAW cannot propagate through the BR. The observed spectra as well as the multiple reflections are consistent with the one-dimensional coupling-of-mode (1D COM) theory^27^ with *r* = 0.01 and the damping, as shown in Fig. 2d,f [See Methods]. The analysis indicates a band gap of Δ*f*_BR_ = 100 MHz in our device.

A well-defined cavity mode can be formed in device B (the same *λ*_BR_ = 0.8 *μ*m) with a small *D* of 4.4 *μ*m shown in Fig. 3a. Inherent cavity spectrum is obtained by using the time-resolved measurement to exclude the EM crosstalk. A short IDT of 10 pairs is used to generate SAW in a broadband, and a long burst wave of about 2500 cycles is used to minimize the spectral broadening of the burst. As shown in Fig. 3b, the spectrum shows sharp peaks, each of which is much narrower (Δ*f*_cav_ = 1.2 MHz for peak I) than the band gap (Δ*f*_BR_ = 100 MHz). Both peak I and II exhibit standing wave nature of the cavity mode in the phase evolution at different PCs, as shown in the right inset for peak I. An approximately *π* phase shift is observed between PC1 to PC3 and PC4 to PC5. This means that the piezoelectric potential has a node located close to the central gate, which proves the realization of a cavity mode [See the left inset].

The above measurements of phonon band gap in the BR and the line width of the cavity mode suggests the cavity finesse, *F* = Δ*f*_BR_/Δ*f*_cav_ ≈ 80, by considering the ratio of the cavity decay time 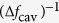 and the time required to reflect SAWs in a BR 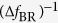. For a given input power or a number of phonons, the cavity is expected to enhance the spontaneous emission as well as stimulated processes by the factor *F*, which is examined with a DQD.

We demonstrate phonon assisted tunneling in a DQD by using the cavity mode. For the time-gated scheme described below, the transport path of Device B is rerouted as shown in Fig. 4a, and the ohmic contact on the left is used as an in-plane gate (IPG). A weakly coupled DQD is formed with G_0−5_ at 30 mK, where thermal excitation of SAW phonons can be neglected. When the detuning *ε* between the ground levels in the left and right dots is varied, a sharp resonant tunneling peak with a width of 16 *μ*eV appears at *ε* = 0, as shown by the bottom trace of Fig. 4b. The spectrum can be fitted with a Lorentzian profile shown by a solid line with a negligible deviation associated with the cotunneling processes at 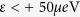. Current associated with the spontaneous phonon emission could not be observed because it was too small in this weak coupling limit. When a continuous wave (CW) at the resonant frequency of 3222 MHz is applied to the IDT, the peak splits into multiple peaks with fine structures in Fig. 4b. In this CW experiment, both photon (the EM crosstalk) and phonon (SAW) contribute to the splitting, as the DQD feels the sum of the oscillating detuning potential 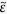. The current spectrum can be explained by phonon and/or photon assisted tunneling with absorption (*n* < 0) or emission (*n* > 0) of 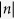 phonons/photons (See insets)^24^,^30^,^31^. They are seen as small current peaks at 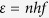 (highlighted by vertical lines) in Fig. 4b even though the linewidth of 16 *μ*eV is slightly larger than the spacing *hf* = 13 *μ*eV. The theory for CW excitation suggests that each peak current should be proportional to the square of the Bessel function of the first kind 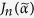 of a normalized amplitude 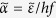. Using a Lorentzian function fitted to the original peak profile at *V*_ex_ = 0, all current profiles can be reproduced accurately by varying 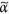 in proportional to *V*_ex_, as shown by solid lines.

Contribution of the phonons can be extracted by employing the time-gated scheme with a burst RF wave *V*_ex_ for exciting phonons and a square voltage pulse *V*_IPG_ for defining the measurement period just after the EM crosstalk has disappeared, as shown in the inset of Fig. 4c and Methods. The left peak, labeled SAW, in the current profile of Fig. 4c measures the phonon-assisted tunneling spectrum driven by phonons accumulated in the cavity. When the carrier frequency *f* is varied as shown, a clear splitting into peaks at 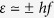 is observed only at the cavity’s resonant frequency (the thick trace at zero offset 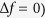. This demonstrates that SAW phonons can be used as an excitation source for a charge qubit in a DQD. The splitting vanishes with a small offset 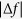 ≳ 1 MHz, consistent with the narrow peak in Fig. 3b. This shows the enhancement of piezoelectric field in the cavity by noting that almost same RF power is introduced to the IDT in the frequency range.

We have proposed and demonstrated a SAW phonon cavity, in which electron-phonon coupling is enhanced. The enhancement should also be applied to the spontaneous emission process, known as the Purcell effect. The spontaneous phonon emission of a charge qubit in a similar GaAs DQD yields the rate 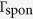 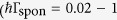 *μ*eV) at the energy spacing of *ε* = 10–30 *μ*eV^2^,^26^. If the DQD emits all phonons into the SAW cavity mode, the ultra-strong coupling regime with 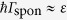 can be reached by enhancing the phonon emission by a factor of 30–500. Such crude estimate encourages further study of cQED with SAW phonons.

## Methods

**The time-resolved phase-sensitive local potential measurement** was performed with a PC as a local potential probe. Its conductance is adjusted in the tunneling regime (≈0.6 *e*^2^/*h*), where it is sensitive to the potential of the barrier. As shown in Fig. 2b, a burst RF wave *V*_ex_ at the carrier frequency *f* is applied to the IDT to generate a burst SAW, which propagates along the sample surface and induces piezoelectric potential ϕ_PC_ at *i*-th PC. This time-dependent potential is demodulated by applying another burst RF wave *V*_det_ at the same *f* on the bias voltage of the PC. The resultant dc current (*I*_*det*_) through the PC is proportional to the convolution of ϕ_PC_ and *V*_det_. Monitoring *I*_det_ while varying the time delay *t*_d_ between *V*_det_ and *V*_ex_ provides a phase-sensitive potential measurement^28^. Generally a burst wave of *N*_B_ cycles repeated for a period of *N*_R_ cycles (*N*_B_ and *N*_R_ in the unit of 1/*f*  ) involves spurious spectra of the spacing *f/N*_R_ in the frequency range of 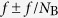. We have carefully chosen *N*_B_ and *N*_R_ such that the spurious spectra does not affect the spectrum of interest. For data in Fig. 2c,e, a SAW packet of about 150 cycles is generated by applying *V*_ex_ with *N*_B_ = 50 to the left IDT with 100 metal pairs. The spatial spreading (≈120 *μ*m) of the packet is shorter than the gap distance *D* = 160 *μ*m to discriminate the right- and left-moving packets. For data in Fig. 3b, a long burst wave of *N*_B_ = 2500 cycles is applied for accumulating phonons in the cavity mode, and is repeated for *N*_R_ = 6000 cycles. The slowly-decaying SAW after the excitation is demodulated with a detection burst of 2500 cycles with delay time *t*_d_ of 3000 cycles of 1/*f*.

**The time-gated phonon-assisted tunneling measurement** is performed with an IPG for time gating. A burst RF wave of 1000 cycles is applied to the 10-pair IDT to accumulate phonons in the cavity. The square wave *V*_IDT_ in the inset of Fig. 3c is designed to measure purely the phonon-assisted tunneling excited by the cavity phonons for the period labeled ‘SAW’ after the excitation burst is turned off. This was done by shifting the spectrum by 100 *μ*eV in detuning with the square wave *V*_IDT_. Corresponding spectrum appears in the current profiles labeled ‘SAW’. The additional spectrum in the profiles labeled ‘EM+SAW’ measure the excitation spectrum from both photons (EM) and phonons (SAW).

**The 1D COM simulation** is performed to deduce the phonon band gap and the device parameters from the time-domain reflectometry in Fig. 2c and the reflection spectrum in Fig. 2e. In this model, the right- and left-moving SAWs are coupled by the scattering matrix with *r*, and the SAWs are coupled to the RF voltage and current in IDT with *K*^2^. A simple reflection coefficient for a continuous-wave SAW can be obtained by the standard frequency domain analysis for the total scattering matrix of the BR. Since a burst wave is employed in this work, the actual spectrum as well as the detector response involves the Fourier series of the burst wave. We took this effect into account in the simulations of Fig. 2d,f. We determined *r* = 0.01 and the damping (25% energy loss at each etching step) to reproduce the experimental data in Fig. 2c,e. The corresponding phonon band gap in an infinite metal array should be 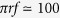 MHz as shown by the arrow labeled Δ*f*_BG_.

**Figure 1:**
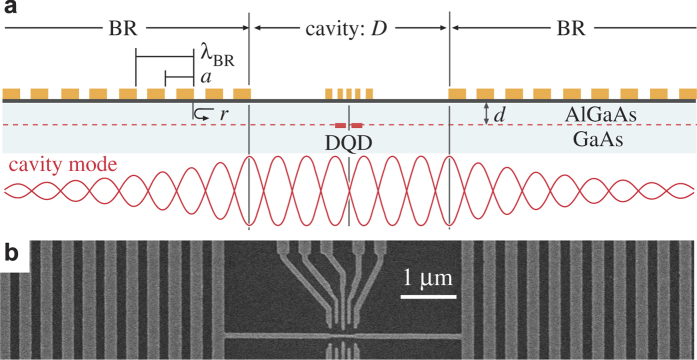
A SAW phonon cavity. (**a**) A schematic of the proposed phonon cavity. Two arrays of BRs for *λ*_BR_ determined by the period *a* are placed at the distance *D*. A DQD is formed at a distance *d* beneath the sample surface. SAWs are reflected at metal edges with a reflection coefficient *r*. Cavity mode is indicated by the standing wave beneath the schematic with a node in the middle of the DQD so maximum electron-phonon coupling can be obtained. (**b**) A scanning electron micrograph (SEM) of a control device with identical design to Device B. Metal layers with the thickness of 10 nm for Ti and 30 nm for Au are patterned. Periodic arrays serve as BRs for forming a phonon cavity and IDT for generating SAWs. Fine metal fingers in the middle of the cavity are used to define point contacts for the phase-sensitive local potential measurements or a DQD for studying phonon assisted tunneling.

**Figure 2:**
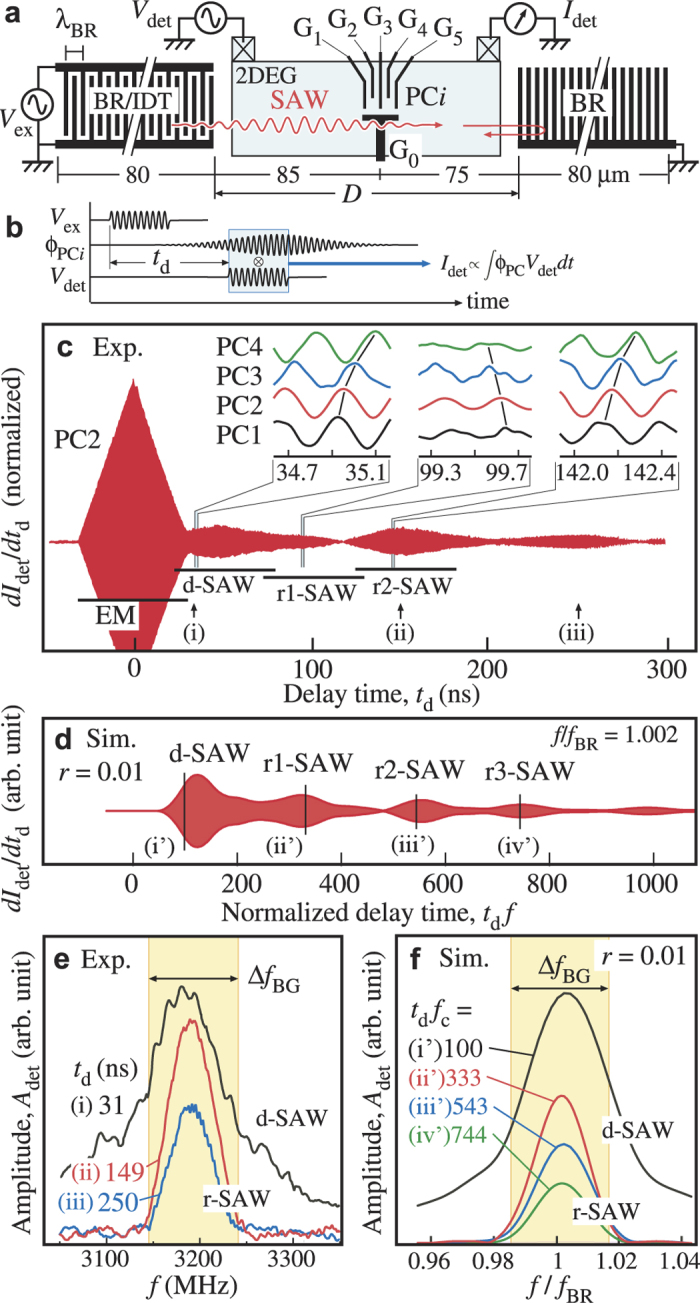
Phonon bandgap in a BR. (**a**) A schematic diagram for Device A used for the study of Bragg reflection. A SAW packet is launched by applying *V*_ex_ to the left IDT. PCs placed in a gap of *D* = 160 *μ*m are used to detect SAWs reflected between the two BRs. (**b**) The pulse pattern used for the Bragg-reflection measurements. When the SAW wave packet generated by *V*_ex_ arrives at the PC, it oscillates the PC’s electric potential as ϕ^PC^. This oscillation can be demodulated by applying *V*_det_ and the measured current *I*_det_ across the PC constitutes the convoluted signal of *V*_det_ and ϕ^PC^, allowing us to extract the SAW signal. (**c**) PC current differentiated with respect to *t*_d_ plotted as a function of delay time at 4 K. EM crosstalk, direct wave packet (d-SAW), 1st and 2nd reflected wave packets (r1- and r2-SAW) can be observed. Inset: Comparisons of the signals measured at *i*-th PC defined by gates G_*i*_ and G_0_. Vertical offsets and black lines are applied to highlight the phase evolution. (**d**) Theoretical calculations of the expected wave packets at PC2 by the 1D COM theory. (**e**,**f**) Frequency dependence of the oscillation amplitudes *A*_det_ in 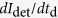 at some delay times (**e**) measured with PC2 at 0.3 K and (**f**) simulated by the 1D COM theory. While the spectrum of the d-SAW packet reflects the Fourier spectrum of the incident wavepacket, that of r-SAW measures the phonon bandgap of the BRs.

**Figure 3:**
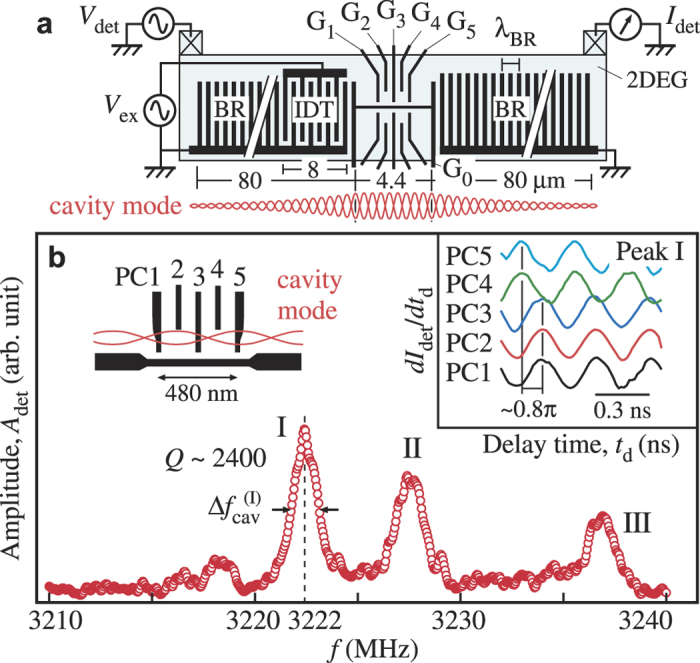
The SAW cavity mode. (**a**) A schematic diagram for device B with circuit diagrams used in this measurement. This device was used to study the formation of SAW cavity modes and the coupling to a charge qubit. G_0_ separates the device into top and bottom channels and the charge qubit is formed in the top channel by activating gates G_1−5_. A small *D* is chosen to enhance the electron-phonon coupling inside the small cavity. (**b**) Frequency dependence of the SAW amplitude at PC2, taken at 30 mK. The major peaks (I & II) exhibit standing wave nature which is shown in the right inset for peak I. Right inset: A comparison of the signals’ phase measured at PC1-5. An approximately *π* phase difference is observed between PC1-3 and PC4-5, which demonstrates the formation of a standing wave inside this device. Left inset: A schematic of the cavity mode with respect to the DQD.

**Figure 4:**
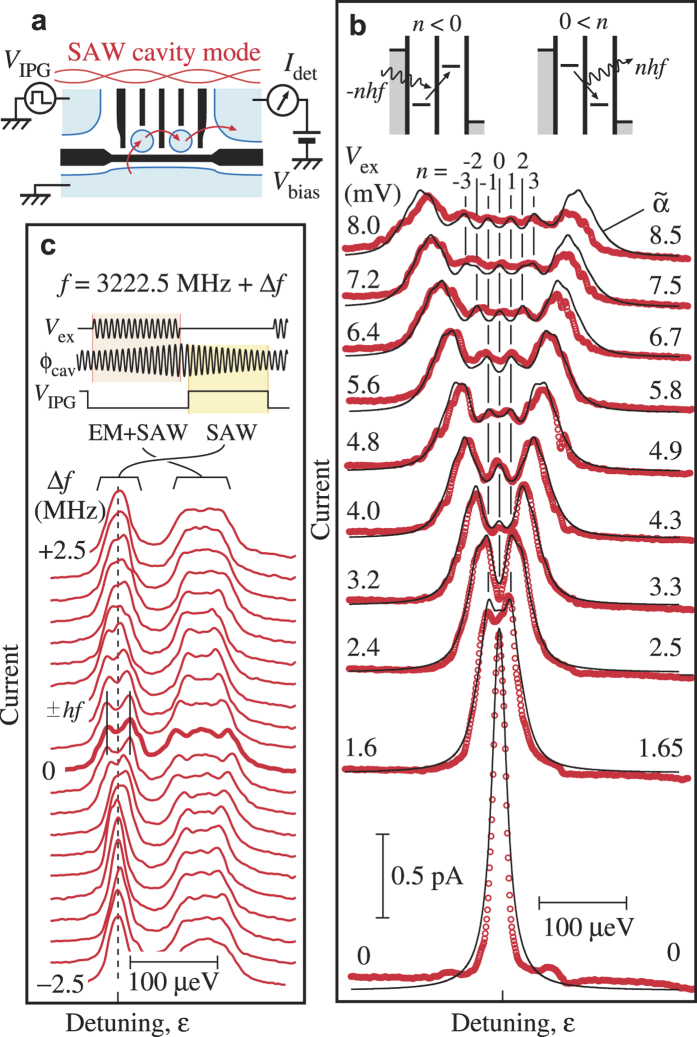
Phonon assisted tunneling in a DQD. (**a**) Schematic of the time-gated measurement setup. The left ohmic contact is used as an in-plane gate to extract solely the phonon assisted tunneling spectrum. Current is re-routed to flow from the bottom channel, through the DQD to the right ohmic contact. (**b**) Excitation voltage dependence of the splitting in resonant tunneling peaks. Phonon/photon assisted tunneling sidebands are observed with separations in the unit of *hf*. Measured data (red circles) can be reproduced by theoretical fits (solid black lines), which suggests emission and absorption of integer numbers of phonons by the DQD. (**c**) Frequency dependence of the current profile under the pulse sequence shown in the inset. Phonon assisted tunneling spectra, labeled SAW, show a splitting of ±*hf* (marked by vertical lines) only at the resonant frequency (Δ*f* = 0). The other spectra, labeled ‘EM+SAW’, are excited by the mixture. The two sets of spectra are separated by the application of the square voltage pulse *V*_IPG_. Data in (**b**,**c**) are taken at 30 mK.

**The multiple peaks in Device B.** Similar 1D COM simulation for Device B suggests only one cavity mode in the band gap. The multiple peaks in Fig. 3b might be caused by the complicated gate patterns with inhomogeneous metal coverage. Since the velocity in the metalized region is reduced by 10% in our device, the resonant frequency should be affected by the wider gate patterns extended from the central region [See SEM micrograph with an identical design in Fig. 1b]. The excess metallized regions of 0.6 *μ*m along the SAW propagation direction are sufficient to explain the spacing between peaks I and II. Two-dimensional mode analysis is desirable to identify multiple modes as well as to design a phononic crystal cavity.

## Additional Information

**How to cite this article**: Chen, J. C. H. *et al.* Enhanced electron-phonon coupling for a semiconductor charge qubit in a surface phonon cavity. *Sci. Rep.* **5**, 15176; doi: 10.1038/srep15176 (2015).
